# Application of situational simulation based on the Mini-CEX and DOPS rating scales in urology clinical training

**DOI:** 10.3389/fmed.2024.1480081

**Published:** 2024-11-26

**Authors:** Ben Xu, Jia-en Zhang, Lin Ye, Chang-wei Yuan

**Affiliations:** Department of Urology, Peking University First Hospital and Institute of Urology, Peking University, Beijing, China

**Keywords:** Mini-CEX score, DOPS score, situational simulation teaching, urology, clinical trainee teaching, satisfaction

## Abstract

**Objective:**

The objective of the study was to investigate the application effect of scenario simulation teaching based on the Mini-Clinical Evaluation Exercise (Mini-CEX) and direct observation of procedural skills (DOPS) rating scales in urology clinical apprenticeship training.

**Materials and methods:**

A total of 32 students from the class of 2015–2017 who completed their traineeship in the Department of Urology of our hospital were selected and divided into a research group and a routine group. Routine teaching was implemented for the trainees in the control group, while situational simulation teaching based on the Mini-CEX and DOPS scoring scales was implemented for the trainees in the research group. The Mini-CEX and DOPS scores and student satisfaction were compared at the time of admission and discharge between the two groups.

**Results:**

At the time of admission, there was no statistically significant difference between the Mini-CEX and DOPS scores of the two groups of trainees (*p* > 0.05). However, at the time of discharge, the Mini-CEX and DOPS scores of the trainees in the study group were 53.21 ± 4.52 and 81.23 ± 3.57, respectively, which were significantly higher than those in the conventional group (*p* < 0.001), and the trainees in the study group’s satisfaction with teaching was (20.11 ± 2.31), which was significantly higher than that of the conventional group (*p* < 0.001).

**Conclusion:**

Implementing scenario simulation teaching based on the Mini-CEX and DOPS rating scales in urology clinical traineeship can significantly improve trainees’ surgical skills while also leading to high levels of satisfaction with the teaching method.

## 1 Introduction

Urology, as a field that focuses on diseases of the genitourinary system, places a strong emphasis on practical skills in clinical practice, and in clinical internship teaching, emphasis is placed on developing practical skills (1). However, relevant studies (2, 3) have shown that traditional teaching methods in medical traineeships, which focus on centralized lectures, free practice, and teacher-centered instructions using videos, images, or text, often lead to students passively receiving information rather than engaging in with it. This approach can result in a disconnect between theoretical knowledge and clinical practice, negatively impacting the quality of teaching. At the same time, the existing apprenticeship assessments rely on theoretical testing and lack situational simulation and other testing methods, making it difficult to effectively assess the clinical decision-making and communication skills of apprenticeship students (4). The Mini-Clinical Evaluation Exercise (Mini-CEX) (5) and direct observation of procedural skills (DOPS) (6), as effective tools for field exercise assessment, can accurately reflect trainees’ on-site decision-making and clinical practice abilities. These two assessment tools have already been applied in gynecology (7) and stomatology (8), yielding positive results. This study was conducted to investigate the effectiveness of scenario-based simulation teaching using the Mini-CEX and DOPS rating scales in urology clinical traineeship.

Although the article was recently compiled and published, its data actually date back to 2018–2019. Based on a literature review, this study appears to be an early exploration of the practical application of the Mini-CEX and DOPS in clinical teaching. The majority of the current literature focuses on studies from 2023 to 2024. This study is likely one of the earliest research efforts on the application of the Mini-CEX and DOPS rating scales in urology clinical traineeships. In addition, this study innovatively applied the scenario-based simulation teaching method alongside the Mini-CEX and DOPS rating scales to achieve a joint and comprehensive application of multiple teaching evaluation tools. Although a recent 2024 study by Priyanka Rai and her co-authors, titled “Assessment of residents in the department of surgery in a tertiary care center using a mini-clinical evaluation exercise,” reported positive results, the study focused on resident physicians who already possess a certain level of basic knowledge and skills in urology. Therefore, the Mini-CEX can be more effectively implemented among resident physicians who have certain basic knowledge and skills. However, to the best of our knowledge, there have been no studies on the use of the Mini-CEX and DOPS rating scales during the internship or traineeship stage in urology.

The final observation indicators of this study were comprehensively assessed from three dimensions: students’ comprehensive clinical reasoning ability, surgical skills, and subjective satisfaction. (1) The Mini-CEX tool is used by clinical teachers to directly observe the clinical diagnosis and treatment processes of students, including their inquiry skills, physical examination skills, clinical interpretation, communication skills, clinical operation abilities, professional attitude, organizational effectiveness, and overall clinical competence. After the observation is completed, any issues found in students’ observations are corrected in a timely manner, and the students are scored using structured table items to provide timely feedback. (2) Compared to the Mini-CEX tool, the DOPS tool focuses more on clinical skill operation, with the teacher directly observing the student’s clinical operation technique, evaluating 11 indicators of a certain skill operation, and providing feedback. (3) A self-assessment questionnaire is used to assess students’ overall satisfaction with the course. In summary, by conducting these three components of research, the dual improvement of students’ knowledge, skills, and satisfaction can be achieved.

## 2 Materials and methods

### 2.1 Basic information

A total of 32 students from the 2015–2017 class of the 8-year basic and clinical undergraduate medical program, who were undergoing traineeship in the Department of Urology of our hospital in 2018–2019, were selected as the study participants. This included 19 male and 13 female students, with seven from the 2015 class, 11 from the 2016 class, and 14 from the 2017 class. All of the trainees were fourth-year undergraduate medical students who had received basic theoretical knowledge of urology but had not yet entered the urology ward and did not have the ability to directly apply their theoretical knowledge to clinical practice. The trainees had just entered the hospital and begun their internship, so they were not yet accustomed to using the Mini-CEX and DOPS for assessments in other departments. Before officially starting their urology internship, the teacher conducted relevant training on the Mini-CEX and DOPS. The sample size included in the experiment referred to the actual number of students who participated in internships and apprenticeships during the author’s tenure as a teaching instructor. According to the principle of matching, the students were divided into two groups: the research group and the conventional group, with 16 students in each group. The basic data of the two groups of students were compared, and the difference was not found to be statistically significant (*p* > 0.05) (See Table 1 for details). All students expressed satisfaction with their participation in this study. None of the students objected to this method of training. All of the methods were carried out in accordance with the relevant guidelines and regulations of the Declaration of Helsinki. All of the experimental protocols were approved by Peking University First Hospital.

**Table 1:** Basic characteristics of the two groups of students.

Group	No.	Sex	Grade
		Male	Female	2015	2016	2017
Test	16	9 (56.3)	7 (43.7)	4 (25.00)	6 (37.50)	6 (37.50)
Control	16	10 (62.5)	6 (37.5)	3 (18.75)	5 (31.25)	8 (50.00)
*χ^2^*		0.130	0.519
*p*		0.719	0.771

### 2.2 Research methods

For the trainees in the control group, conventional teaching was implemented, according to the syllabus, through collective lectures and physician-led teaching. The trainees were taught about occurrence mechanisms, treatment methods, precautions, and other aspects of urology-related diseases. During the course of their clinical work, the trainees were encouraged to take the initiative in participating in the treatment of related diseases and were guided to understand their job responsibilities, as well as the relevant knowledge and skills they needed to master.

Scenario simulation teaching based on the Mini-CEX and DOPS rating scale was implemented for the trainees in the study group. The scenario exercises were designed around situations encountered in clinical practice as a framework (9).

A total of two scenarios were developed for simulation teaching: Case I involved Abdominal pain suspected to be caused by hemorrhagic shock with kidney injury, and Case II involved Abdominal pain suspected to be caused by acute urinary retention with prostatic hyperplasia. Each scenario involved a progressive examination of the following five aspects: 1. Collecion of patient history and physical examination; 2. documentation of medical records; 3. diagnosis and differential diagnosis, along with the formulation of a treatment plan; 4. doctor–patient communication and preoperative discussion; and 5. clinical practice (Case 1: incision and suture, deep vascular knotting, and tension knots and Case 2: urinary catheterization).

### 2.3 Scenario simulation

The simulation included the following components:

① Theoretical explanation: The instructor provided an explanation of theoretical knowledge to improve the trainees’ understanding of the disease and its associated precautions. ② Scenario exercise background introduction: This included the patient’s basic information, reason for admission to the hospital, underlying conditions, treatment history, current vital signs, emotional state, and accompanying personnel, among other details (10). ③ Scoring criteria: The Mini-CEX and DOPS scores were provided to the trainees, and they were informed that the simulation exercise would serve as the basis for assessment. ④ Live simulation demonstration: The instructor role-played as the patient. The trainee students were randomly divided into two groups, each responsible for receiving a case. A group leader was assigned who was primarily responsible for answering questions, while other group members contributed to the answers. The members of the group could discuss any disagreements and ultimately reach a consensus. ⑤ The instructor referred to the Mini-CEX and the DOPS scoring scale content, and the trainee students were asked to refer to the scores of the Mini-CEX and DOPS scales. The DOPS scoring scale was used to score the simulation-based teaching. ⑥ Summary and feedback: A collective discussion was held, where the trainees were encouraged to share their feelings about the exercise and any issues observed in the exercise process of other groups. The students also discussed the problems and precautions in the lesson plan and offered suggestions and feedback.; ⑦ Summary and rectification: The lead instructor addressed the common issues based on the performance of the each group and provided corrective feedback.

### 2.4 Observation indicators

#### 2.4.1 Mini-CEX score

When the two groups of trainees were admitted to and discharged from the department, the Mini-CEX scale was used to evaluate their clinical exercises (11). This included the following seven dimensions: medical questioning skills, organizational effectiveness, humanitarianism, clinical judgment, physical examination, communication skills, and overall performance. The scores for each dimension ranged from 1 to 9 points. A score of ≤3 points was considered unqualified, 4–6 points was considered qualified, and ≥ 7 points was considered excellent. The total score was 63 points, with a score of ≥38 considered qualified and a score of ≥49 considered excellent.

#### 2.4.2 DOPS score

At the time of admission and discharge of the two groups of trainees, the DOPS scale was used to evaluate their clinical surgical skills (11). This scale included the following 11 dimensions: understanding of the indications and relevant anatomical knowledge, obtaining the consent of the patient and the family, preoperative preparation, appropriate analgesia or sedation, surgical ability, aseptic technique, timely request for help, postoperative treatment, communication skills, humanistic care, and overall performance. The scores for each dimension ranged from 1 to 9 points, with a rating of ≤3 points considered unqualified, 4–6 points considered qualified, and ≥ 7 points considered excellent. The total score was 99 points, with ratings of ≥44 points considered qualified and ratings of ≥77 points considered excellent.

#### 2.4.3 Teaching satisfaction

When the trainees were discharged from the department, their teaching satisfaction was evaluated using a self-made satisfaction questionnaire, which included five dimensions—learning interest, teacher–student interaction, teamwork, doctor–patient communication, and independent learning. Each dimension was scored on a scale of 1 to 5 points, with a possible total of 25 points. The scores were positively correlated with trainees’ satisfaction with the teaching.

### 2.5 Data processing

SPSS 27.0 statistical software was used to enter the data into the database. The normally distributed measurements were expressed as mean ± standard deviation (*x–±s*), and a *t*-test was used for comparisons between groups. The count data were expressed as percentages (%), and the *χ*^2^ test was applied. A *p*-value of <0.05 was considered statistically significant.

## 3 Results

### 3.1 Mini-CEX score

At the time of admission, there was no statistically significant difference between the Mini-CEX scores of the two groups (*p* > 0.05) (Table 1). At the time of discharge, the Mini-CEX scores of the trainees in the study group were significantly higher (53.21 ± 4.52) compared to those in the conventional group (43.68 ± 2.36) (*p* < 0.001), as shown in Table 2. From Table 2, it can be seen that the effect sizes and Cohen’s d for the *t*-test indicated no significant difference in the mean scores between the two groups at the time of admission (Cohen’s *d* < 0.2). However, the difference at the time of discharge was quite significant (Cohen’s *d* > 0.8).

**Table 2:** Comparison of the Mini-CEX scores at different times between the two groups of trainees (scores).

Time	Group	Questioning Skills	Organizational Effectiveness	Humanism	Clinical Judgment	Physical Examination	Communication Skills	Overall Performance	Scores
Entry	Test	4.21 ± 1.10	4.42 ± 1.17	4.32 ± 1.12	4.43 ± 1.19	4.39 ± 1.35	4.69 ± 1.24	4.42 ± 1.25	31.06 ± 2.96
	Control	4.23 ± 1.34	4.33 ± 1.25	4.47 ± 1.22	4.53 ± 1.17	4.31 ± 1.27	4.75 ± 1.31	4.36 ± 1.07	30.89 ± 3.06
	*t*	0.046	0.210	0.362	0.240	0.173	0.133	0.146	0.160
	*p*	0.964	0.835	0.720	0.812	0.864	0.895	0.885	0.874
	Cohen’s d	0.016	0.074	0.128	0.085	0.061	0.047	0.052	0.056
	Effect sizes	0.008	0.037	0.064	0.042	0.031	0.024	0.026	0.028
Exit	Test	7.42 ± 1.21	7.55 ± 1.12	7.36 ± 0.98	7.47 ± 0.88	7.37 ± 0.89	7.86 ± 0.69	7.68 ± 0.86	53.21 ± 4.52
	Control	6.16 ± 1.56	6.24 ± 1.49	6.21 ± 1.23	6.01 ± 0.52	6.26 ± 0.68	6.09 ± 0.67	6.13 ± 0.87	43.68 ± 2.36
	*t*	2.553	2.811	2.925	5.713	3.964	7.361	5.068	7.476
	*p*	0.016	0.009	0.007	<0.001	<0.001	<0.001	<0.001	<0.001
	Cohen’s d	0.903	0.994	1.034	2.020	1.402	2.603	1.792	2.643
	Effect sizes	0.411	0.445	0.459	0.711	0.574	0.793	0.667	0.797

### 3.2 DOPS score

At the time of admission, there was no statistically significant difference between the DOPS scores of the two groups of trainees (*p* > 0.05). The effect sizes and Cohen’s d for the *t*-test indicated that the difference in the mean was not significant (Table 3).

**Table 3:** Comparison of the DOPS scores at the time of admission between the two groups of trainees (scores).

Dimension				Entry		
	Test	Control	*t*	*p*	Cohen’s *d*	Effect sizes
1. Knowledge of indications and related anatomy	3.47 ± 1.31	3.23 ± 1.06	0.570	0.573	0.201	0.100
2. Obtain consent from the patient and family	3.57 ± 1.38	3.68 ± 1.42	0.222	0.826	0.079	0.039
3. Pre-operation preparation	3.20 ± 1.03	3.17 ± 1.08	0.080	0.936	0.028	0.014
4. Appropriate pain relief or sedation	3.31 ± 1.06	3.40 ± 1.13	0.232	0.818	0.082	0.041
5. Operational capability	3.70 ± 1.34	3.89 ± 1.41	0.391	0.699	0.138	0.069
6. Aseptic technology	3.57 ± 1.25	3.62 ± 1.26	0.113	0.911	0.040	0.020
7. Demanding help at the right time	3.61 ± 1.38	3.48 ± 1.34	0.270	0.789	0.096	0.048
8. Postoperative treatment	3.13 ± 1.05	3.24 ± 1.09	0.291	0.773	0.103	0.051
9. Communication skills	3.38 ± 1.01	3.42 ± 1.08	0.108	0.915	0.038	0.019
10. Humanistic care	3.53 ± 1.28	3.46 ± 1.24	0.157	0.876	0.056	0.028
11. Overall performance	3.47 ± 1.19	3.39 ± 1.25	0.185	0.854	0.066	0.033
Total scores	37.89 ± 3.21	37.56 ± 3.45	0.280	0.781	0.099	0.049

At the time of discharge, the DOPS score of the trainees in the study group was significantly higher (81.23 ± 3.57) compared to the conventional group (59.12 ± 3.52) (*p* < 0.001). The effect sizes and Cohen’s d for the *t*-test indicated that the difference in the mean was quite significant (Table 4).

**Table 4:** Comparison of the DOPS scores at the time of discharge between the two groups of trainees (scores).

Dimension	Exit					
	Test	Control	*t*	*p*	Cohen’s d	Effect sizes
1. Knowledge of indications and related anatomy	7.35 ± 1.31	5.69 ± 1.25	3.667	0.001	1.297	0.544
2. Obtain consent from the patient and family	7.53 ± 1.25	6.12 ± 1.07	3.428	0.002	1.212	0.518
3. Pre-operation preparation	7.46 ± 1.03	5.89 ± 1.32	3.751	0.001	1.326	0.553
4. Appropriate pain relief or sedation	7.35 ± 1.32	5.79 ± 1.05	3.700	0.001	1.308	0.547
5. Operational capability	7.49 ± 1.28	5.83 ± 1.25	3.711	0.001	1.312	0.549
6. Aseptic technology	7.52 ± 0.97	5.97 ± 1.22	3.978	<0.001	1.406	0.575
7. Demanding help at the right time	7.26 ± 0.86	6.02 ± 0.95	3.871	0.001	1.368	0.565
8. Postoperative treatment	7.34 ± 1.21	5.86 ± 1.32	3.306	0.002	1.169	0.505
9. Communication skills	7.46 ± 1.05	5.76 ± 1.27	4.127	<0.001	1.459	0.589
10. Humanistic care	7.62 ± 1.23	6.03 ± 1.31	3.539	0.001	1.251	0.530
11. Overall performance	7.25 ± 1.42	5.89 ± 1.24	2.886	0.007	1.020	0.454
Total scores	81.23 ± 3.57	59.12 ± 3.52	17.640	<0.001	6.237	0.952

### 3.3 Satisfaction with teaching

The satisfaction score of the trainees with teaching in the study group was significantly higher (20.11 ± 2.31) compared to the conventional group (14.21 ± 2.58) (*p* < 0.001), as shown in Table 5. In addition, the effect sizes and Cohen’s d for the *t*-test indicated that the difference in the mean was quite significant.

**Table 5:** Comparison of the teaching satisfaction level of the two groups of trainees (scores).

Group	Interest in learning	Teacher–student interaction	Teamwork	Patient–doctor communication	Independent study	Total
Test	4.03 ± 0.65	4.13 ± 0.56	3.98 ± 0.65	3.95 ± 0.57	4.09 ± 0.61	20.11 ± 2.31
Control	2.59 ± 0.69	2.81 ± 0.70	2.86 ± 0.75	2.84 ± 0.62	2.97 ± 0.58	14.21 ± 2.58
*t*	6.076	5.890	4.514	5.272	5.322	6.815
*p*	<0.001	<0.001	<0.001	<0.001	<0.001	<0.001
Cohen’s d	2.148	2.082	1.596	1.864	1.882	2.409
Effect sizes	0.732	0.721	0.624	0.682	0.685	0.769

## 4 Discussion

Urology is a practice-oriented surgical discipline, and urology clinical apprenticeship is a mandatory training process for medical students before they begin their independent clinical work. Its purpose is to gradually develop the clinical competence of trainee doctors. Although trainee physicians are still in the early stages of acquiring basic clinical knowledge and skills, supervising physicians should establish a solid foundation for their development into excellent and qualified residents in the future through a scientific and reasonable trainee curriculum. This will enable them to acquire the six core competencies of professionalism, knowledge and skills, patient care, communication and cooperation, teaching ability, and lifelong learning (12).

Traditional urology traineeships focus purely on developing the theoretical knowledge of trainee physicians. However, due to the complexity of patients’ conditions and individual situations in clinical practice, the majority of trainee physicians have difficulty in rapidly integrating theory and practice, which affects the efficiency and quality of their clinical work (13). Moreover, traditional urology traineeship teaching is insufficient for teaching and evaluating clinical thinking, basic skills, professionalism, patient care, and communication and cooperation. Traditional evaluation of trainee performance at discharge is based on summative assessments. However, traineeship is different from classroom-based theoretical learning. Although summative evaluation can assess students’ ability to some extent, it lacks a quantitative, objective, and timely assessment and feedback system during the training process. Furthermore, the observation and assessment of trainee physicians’ clinical reasoning and communication skills should be carried out mainly through the process of evaluation. Relying on simple, dogmatic final evaluations may even have a negative impact on trainee physicians’ clinical reasoning and communication skills. Such evaluations may even have a negative impact on the independent learning and performance of trainee physicians. Zhou LQ (14) pointed out that although the traditional apprenticeship process can assess a trainee’s hands-on abilities, it lacks objective and timely assessments, which, in turn, affects the quality of the apprenticeship.

The American Board of Internal Medicine developed the Mini-CEX in 1994 to assess comprehensive clinical skill methods. Many studies have reported the effectiveness of the Mini-CEX in assessing clinical skills (15). However, this method primarily focuses on the assessment of skills across all aspects of patient care in clinical practice. For this reason, the Royal College of Physicians developed the DOPS in 2002, with a particular focus on the assessment of technical skills. It is now widely used in the clinical field (16). In the present study, the application of these two assessment tools was supplemented by a clinical scenario simulation. This allowed the trainee physicians to engage in a more clinically realistic scenario that closely mirrored a real-life clinical situation while ensuring medical safety. In simulated real-life scenarios, trainee physicians can be better equipped in terms of professionalism, patient care, communication and cooperation, and other comprehensive non-skilled competencies (17).

The Mini-CEX and DOPS are also used for the assessment of clinical skills. At the same time, the Mini-CEX and DOPS are work scenario-based assessment tools, and their effectiveness can be better utilized in scenarios that simulate real clinical situations as closely as possible. With the development of related research, the Mini-CEX and DOPS rating scales have been gradually applied to resident training (18), yielding positive results. However, their use in the training and teaching of lower-level trainee physicians is still poorly reported. Our research team innovatively applied the Mini-CEX and DOPS rating scales earlier in trainee teaching, aiming to promote the training and development of the trainee physicians’ clinical competence at an earlier stage.

The use of scenario simulations rather than real cases for teaching and assessment is mainly due to concerns related to clinical diagnosis, treatment safety, and protection of patients (19). For inexperienced trainee physicians, unfamiliarity with procedures and communication barriers between doctors and patients may lead to medical disputes. Moreover, the large number of trainee physicians makes it difficult to arrange real cases for each trainee physician to be assessed effectively. Conducting assessments of multiple trainee physicians on a single patient is also challenging, particularly because some invasive procedures are impossible to repeat on the same patient several times. Assessments based on clinical scenario simulations help avoid the above-mentioned risks, allowing candidates to go through the process of training and assessment while gaining a better understanding of the entire diagnosis and treatment process. This approach provides a more solid foundation for future interactions with patients in clinical practice. In the scenario simulation assessment, the use of fixed scenarios, role-playing, and faculty-guided simulations ensures that the teaching staff are thoroughly familiar with the syllabus, the objectives of the simulation, and the assessment criteria., This ensures that each trainee physician receives more consistent training, leading to final results with a higher degree of credibility.

The key difference between the scenario-based assessment tool and the traditional grading scale is timely feedback and communication. In this study, the researchers emphasized providing feedback on the overall performance of the trainees. During the assessment process, the researchers provided timely feedback to the trainees based on the specific assessment items and scenarios in line with the evaluation criteria of the Mini-CEX and DOPS. They identified the weaknesses of the trainees, corrected them immediately, and encouraged the trainees to make continuous improvement. This approach significantly increased the satisfaction of the trainees at the end of the rotations. In addition, feedback on the weaknesses of the trainees was shared with subsequent teaching physicians so that they could guide other teaching physicians in the subsequent training of the trainees. Feedback on the weaknesses of the trainee physicians was also shared with subsequent trainers so that they could guide other trainers in providing more targeted guidance during the subsequent training session.

The results of this study showed that scenario simulation teaching based on the Mini-CEX and DOPS rating scales can improve the satisfaction of trainee physicians at the end of the rotation. When the trainees with higher levels of disease management and practical skills were discharged from the department, the Mini-CEX and DOPS scores of the trainees in the research group were 53.21 ± 4.52 and 81.23 ± 3.57, respectively, which were significantly higher than those of the conventional group (*p* < 0.001). This finding is in line with the results of the study by Jiang SJ (20). The reasons for this are as follows: The Mini-CEX and DOPS rating scales can more accurately assess trainees’ practical abilities, and scenario simulation exercises based on these scales can more realistically reflect trainees’ clinical skills compared to traditional theoretical teaching. In this study, actual cases were used as scenario blueprints, and self-study was facilitated through group exercises, which helped improve the clinical participation and practical abilities of the trainees (21, 22). Meanwhile, after the blueprint was completed based on group members, the two scales and scoring criteria were provided to the group members, which improved their understanding of clinical precautions. This approach, which included self-learning and rehearsal, helped improve their self-directed learning abilities (23). In addition, Xu TH (24) noted in their study that the use of the Mini-CEX and DOPS scoring scales when conducting scenario rehearsals can significantly improve the scientific rigor and feasibility of the rehearsals, which is important for improving the quality of the training.

In addition, the results of this study showed that scenario simulation teaching based on the Mini-CEX and DOPS rating scales could improve trainees’ satisfaction with the exercises. The trainees in the study group rated their satisfaction with the teaching significantly higher than those in the conventional group (*p* < 0.001), suggesting that the model was accepted by the trainees. The are several reasons for this, including the fact that compared to conventional lectures, the model can increase the trainee’s level of clinical participation (25, 26) and effectively improve their clinical practice. At the same time, in this model, trainees are able to put forward their own ideas and fully express their preferences after the exercises. In addition, compared to conventional lectures centered on the instructor, this model pays more attention to trainees’ attitudes and motivation, which, in turn, helps improve their satisfaction (27).

There have been several studies related to the application of the Mini-CEX and DOPS scales globally. Li ZY et al. proposed that the Advanced Life Support in Obstetrics teaching has an ideal effect on the standardization training of residents of obstetrics, highlighting the prospect of active in-depth research and broader application of the DOPS and Mini-CEX scales (28). Khajehpour et al. (29) designed and implemented a mixed OMMID midwifery professional competence test using the DOPS and Mini-CEX. Alkalash et al. (30) insisted that workplace-based assessment using the Mini-CEX and DOPS demonstrated its ability to improve clinical knowledge and skills among family medicine postgraduates, who became motivated to repeat the process to improve their clinical performance and reduce the stress associated with final summative and objective structured clinical examinations. Similar to our research, Luo P et al. also focused on fourth-year medical students before they entered the clinical environment by using the DOPS and Mini-CEX rating scales. Both tools, with immediate feedback, could significantly enhance surgical clerks’ self-confidence and their clinical competence (31). Schwitz et al. (32) emphasized that all users must be trained in the use of these tools. In particular, it is important to provide immediate and specific feedback that identifies opportunities for improvement and establsihes achievable learning goals. Documentation should be user-friendly and provide an overview of the learning process when using the DOPS and Mini-CEX. In developing countries such as India, the DOPS and Mini-CEX scales are also increasingly being used to assess trainees through direct observation to shape their learning. These tools are feasible, acceptable, and effective in improving the overall learning and competency of postgraduates (33). Moreover, in addition to medical students, the DOPS and Mini-CEX are also effective for nursing students, thereby making these tools suitable for use in the training of students across various medical specialties, not limited to the training of clinical doctors (34). Similar to our research methodology, Xu et al. (35) and Yamauchi et al. (36) also applied the peer role-playing method in clinical teaching using the Mini-CEX to evaluate the clinical skill performance of pediatric trainees and musculoskeletal physical examinations. They found that the role-playing method effectively improved clinical skills, developed clinical communication skills, and enhanced the application of medical knowledge in a simulated medical environment. Peer role-playing as a low-fidelity simulation and practical educational opportunity can enable educators to refine the competency of medical students in physical examinations, clinical reasoning, and diagnosis in a clinical setting.

There are also some limitations to this study. Firstly, the current study was conducted independently by the researchers, and determining whether standardized and uniform training can be achieved among all teaching physicians will require more effort and time. In addition, the teaching physicians’ understanding of the scenario simulation and their overall performance might have also affected the results of the assessment. Secondly, this study did not use a summative assessment as the endpoint of the observation, which is undoubtedly of utmost importance. We cannot yet accurately determine when this summative assessment should occur in relation to the end of the study and what format and content it should include. We must recognize that these aspects are notoriously difficult to assess with a written summative assessment. Although trainees’ satisfaction improved, there was no effective evaluation of whether their basic clinical knowledge and basic skills, as well as clinical thinking, improved. We speculated that the effect of the scenario-based simulation might just have improved the students’ performance on the Mini-CEX and DOPS scales, without necessarily fostering deep learning of the subject, which will need to be addressed in future studies. Thirdly, there was some bias in the enrollment population of this study as it included both trainee students from basic medical specialties and trainee students from clinical medical specialties. Although both groups received the same basic clinical theory teaching, their differing interests might have led to varying expectations and levels of satisfaction with the curriculum, based on their future career choices.

## 5 Conclusion

In conclusion, the implementation of scenario simulation teaching based on the Mini-CEX and DOPS rating scales in urology clinical traineeship can significantly improve trainees’ clinical surgical skills and examination results at the time of discharge while also leading to high levels of satisfaction with the teaching method.

## Data Availability

The raw data supporting the conclusions of this article will be made available by the authors, without undue reservation.
